# Bile Salt Hydrolase Degrades β-Lactam Antibiotics and Confers Antibiotic Resistance on *Lactobacillus paragasseri*

**DOI:** 10.3389/fmicb.2022.858263

**Published:** 2022-06-06

**Authors:** Hiroyuki Kusada, Masanori Arita, Masanori Tohno, Hideyuki Tamaki

**Affiliations:** ^1^Bioproduction Research Institute, National Institute of Advanced Industrial Science and Technology, Tsukuba, Japan; ^2^Bioinformation and DDBJ Center, National Institute of Genetics, Mishima, Japan; ^3^Research Center of Genetic Resources, Core Technology Research Headquarters, National Agriculture and Food Research Organization, Tsukuba, Japan; ^4^Institute of Livestock and Grassland Science, National Agriculture and Food Research Organization, Nasushiobara, Japan; ^5^Faculty of Life and Environmental Sciences, University of Tsukuba, Tsukuba, Japan

**Keywords:** antibiotic resistance, bile salt hydrolase, *Lactobacillus paragasseri*, Ntn-hydrolase family protein, penicillin acylase

## Abstract

Bile salt hydrolase (BSH) is a well-characterized probiotic enzyme associated with bile detoxification and colonization of lactic acid bacteria in the human gastrointestinal tract. Here, we isolated a putative BSH (LpBSH) from the probiotic bacterium *Lactobacillus paragasseri* JCM 5343^T^ and demonstrated its bifunctional activity that allows it to degrade not only bile salts but also the antibiotic (penicillin). Although antibiotic resistance and bile detoxification have been separately recognized as different microbial functions, our findings suggest that bifunctional BSHs simultaneously confer ecological advantages to host gut bacteria to improve their survival in the mammalian intestine by attaining a high resistance to bile salts and β-lactams. Strain JCM 5343^T^ showed resistance to both bile salts and β-lactam antibiotics, suggesting that LpBSH may be involved in this multi-resistance of the strain. We further verified that such bifunctional enzymes were broadly distributed among the phylogeny, suggesting that the bifunctionality may be conserved in other BSHs of gut bacteria. This study revealed the physiological role and phylogenetic diversity of bifunctional enzymes degrading bile salts and β-lactams in gut bacteria. Furthermore, our findings suggest that the hitherto-overlooked penicillin-degrading activity of penicillin acylase could be a potential new target for the probiotic function of gut bacteria.

## Introduction

Bile salt hydrolase (BSH), also known as choloylglycine hydrolase (EC 3.5.1.24), is a well-known enzyme responsible for hydrolyzing glycine/taurine-conjugated bile salts produced by mammalian digestive tracts (Begley et al., 2006). To date, BSH activity has been found in intestinal microorganisms, particularly in probiotic lactic acid bacteria (e.g., *Lactobacillus* spp.) (Begley et al., 2006; Jones et al., 2008). BSH enzymes play a crucial role in bile detoxification, thereby improving the survivability of host probiotic bacteria within the intestinal environment (Ruiz et al., 2013). Therefore, BSH activity has been widely recognized as a representative probiotic biomarker (Rani et al., 2017).

Penicillin acylase (PA, EC 3.5.1.11) has been commonly used worldwide in the pharmaceutical industry because of its role in catalyzing the hydrolysis of the amide bond of β-lactam antibiotics yielding 6-aminopenicillanic acid (6-APA), an essential intermediate compound in the synthesis of semi-synthetic penicillin compounds (Avinash et al., 2016). Therefore, PA has long been considered an important industrial enzyme rather than an antibiotic resistance enzyme. Indeed, only a few studies in the 1960s (English et al., 1960; Holt and Stewart, 1964) and our recent research (Kusada et al., 2017) highlighted the role of PA in bacterial resistance to β-lactam antibiotics.

Notably, BSH enzymes are structurally and phylogenetically related to PA enzymes (Kumar et al., 2006). Both these enzymes belong to the N-terminal nucleophile (Ntn) hydrolase superfamily (Oinonen and Rouvinen, 2000), and the N-terminal cysteine residue functions as a catalytic nucleophile for the enzymatic activity. Hence, we postulate that the intestinal lactic acid bacteria-derived BSH enzyme could also function as a PA enzyme, and such a bifunctional enzyme would confer antibiotic resistance and bile detoxification to the host bacteria. To date, a well-known PA enzyme (BsPVA) from *Lysinibacillus sphaericus* (formerly *Bacillus sphaericus*) ATCC 14577 has been reported to hydrolyze taurocholic acid (TCA), one of the major conjugated bile salts in the human gut (Daly et al., 2021), while two experimentally characterized BSH enzymes (LgBSH from *Lactobacillus gasseri* FR4 and CpBSH from *Clostridium perfringens* 13) can catalyze penicillin as just one of the substrates (Rossocha et al., 2005; Rani et al., 2017). However, the physiological role and phylogenetic diversity of these bifunctional enzymes showing both BSH and PA activity have not yet been well understood.

To verify our hypothesis, we isolated a candidate gene encoding BSH from *Lactobacillus paragasseri* JCM 5343^T^, a probiotic lactic acid bacterium derived from human feces (Tanizawa et al., 2018), and characterized its enzymatic function associated with BSH and PA activity. We further investigated the phylogenetic diversity of bifunctional enzymes in intestinal microorganisms.

## Materials and Methods

### Bacterial Strains and Culture Condition

The lactic acid bacterium, *Lactobacillus paragasseri* strain JCM 5343^T^, was obtained from the Japan Collection of Microorganisms (RIKEN BioResource Research Center, Ibaraki, Japan). This strain was recently identified as a sister taxon of *Lactobacillus gasseri* based on whole-genome sequence analyses (Tanizawa et al., 2018). Strain JCM 5343^T^ was cultivated in Gifu anaerobic medium (GAM, Nissui Pharmaceutical Co., Ltd., Tokyo, Japan) or de-Man-Rogosa-Sharpe medium (MRS, Difco Laboratories, Detroit, MI, United States) with headspace gas of N_2_/CO_2_ (80:20, v/v) at 37°C under anaerobic conditions. *Escherichia coli* strains DH5α (GMbiolab, Taichung, Taiwan) and Origami™ 2 (DE3) (Novagen, Madison, WI, United States) were used as the host strains for gene cloning and gene expression experiments, respectively. *E. coli* strains were cultured on Luria-Bertani (LB) agar or LB broth supplemented with 100 μg/ml ampicillin (Sigma-Aldrich, Saint Louis, MO, United States) at 37°C.

### Cloning and Heterologous Expression of a Putative BSH Gene

The NCBI BLAST program,^1^ UniProt BLAST tool,^2^ InterProScan sequence search program,^3^ and Pfam^4^ were used for sequence analyses and homology search against the complete genome sequence of strain JCM 5343^T^ (accession number AP018549) obtained in our previous study (Tanizawa et al., 2018). The presence of signal peptides was predicted using the SignalP-5.0 server.^5^ A bile salt hydrolase candidate gene (designated as *lpBSH*, locus tag: LpgJCM5343_00530) was amplified using PrimeSTAR HS DNA Polymerase (TaKaRa, Tokyo, Japan) using the following primer set: 5′-GGAATTCCATATGTGTACTGGTTTAAGATTTACTGATGA-3′ (*Nde*I site underlined) and 5′-CGGAATTCATAAGTAATTA GCTTATCGGCGTT-3′ (*Eco*RI site underlined). PCR amplification was performed with an initial denaturation at 98°C for 5 min, followed by 40 cycles at 98°C for 10 s and 68°C for 1 min. Gene cloning, overexpression, and protein purification were performed as described in our previous study with slight modifications (Kusada et al., 2017). Briefly, the PCR product was sub-cloned into a cold shock expression vector pCold II (TaKaRa), and the resulting plasmid was transformed into *E. coli* Origami™ 2 (DE3) competent cells. The *E. coli* strain was cultured in LB broth supplemented with 100 μg/m ampicillin at 37°C until an optical density (OD_600_) of approximately 0.4–0.6 was attained. After adding isopropyl-β-D-thiogalactopyranoside (IPTG, Nacalai Tesque Inc., Kyoto, Japan) at a final concentration of 100 μM to the culture medium, the *E. coli* cells were incubated at 15°C overnight with shaking. The cells were harvested by centrifugation at 5,800 × *g* for 10 min, suspended in buffer (10 mM Tris, 150 mM NaCl, 5% glycerol, 5 mM imidazole, pH 7.5), and disrupted for 5 min via sonication using an ultrasonic disintegrator (Branson Sonifier 250, Branson, Danbury, CT, United States) in an ice-water bath. The cell debris was removed by centrifugation (5,800 × *g* for 10 min), and the resulting supernatant was further applied to HIS-Select Nickel Affinity Gel (Sigma-Aldrich). According to the manufacturer’s instructions, the His_6_-tagged recombinant protein was washed and eluted using an imidazole-containing buffer. The eluted solution was then dialyzed with a semipermeable membrane (Spectra/Por 3 membrane MWCO: 3,500, Repligen, Waltham, MA, United States) to remove imidazole and concentrated using VIVASPIN 500 concentrators (MWCO: 30,000 PES membrane, Sartorius, Göttingen, Germany). The purified His_6_-LpBSH was treated with a premix sample buffer (Nacalai Tesque), heat-denatured at 95°C for 5 min, and analyzed by sodium dodecyl sulfate-polyacrylamide gel electrophoresis (SDS-PAGE) using 12% Mini-PROTEAN TGX precast polyacrylamide gel (Bio-Rad, Hercules, CA, United States). Protein bands were stained with Bio-Safe™ Coomassie Blue G-250 (Bio-Rad). Based on SDS-PAGE analysis, the molecular weight of the purified His_6_-LpBSH protein was approximately 35.0 kDa in size (Supplementary Figure 1), which corresponded to the molecular masses predicted based on its amino acid sequences (316 amino acids).

### Penicillin Acylase Activity

The penicillin acylase activity of the purified protein was determined as described previously with slight modifications (Kusada et al., 2017; Yasutake et al., 2017). Briefly, the purified LpBSH protein was mixed with 10 mM penicillin G solution and incubated at 37°C. As a negative control, penicillin G solution was mixed with a buffer (no enzyme control). After incubation for 12 h, the digestion mixtures were extracted with equal volumes of ethyl acetate three times, and the organic phase was then evaporated to dryness using a vacuum evaporator (EYELA, Tokyo, Japan). The re-dissolved samples in methanol were introduced onto a SHIMADZU GCMS-QP5050 system (Shimadzu Co., Ltd., Kyoto, Japan) equipped with a DB-5 capillary column (30 m × 0.25 mm, 0.25 μm film thickness; Agilent Technologies, Palo Alto, CA, United States).

### Bile Salt Hydrolase Activity

The bile salt hydrolyzing activity of the purified protein was evaluated as described previously (Allain et al., 2018b; Kusada et al., 2021). The purified LpBSH (100 μg/100 μl) was mixed with eight different conjugated bile salts (each 0.24 mg/100 μl) and incubated at 37°C. Each bile salt solution treated with buffer (instead of LpBSH) was used as a negative control. The bile salt hydrolysis reaction was stopped by adding 200 μl of 15% trichloroacetic acid (FUJIFILM Wako Pure Chemical Corporation, Osaka, Japan), and the proteins were precipitated by centrifugation at 10,000 × *g* for 15 min. The supernatant (80 μl) was then mixed with 680 μl of 0.3 M borate buffer with 1% SDS (pH 9.5) and 80 μl of 0.3% 2,4,6-trinitrobenzenesulfonic acid solution (Tokyo Kasei Kogyo Co., Ltd., Tokyo, Japan). This mixture was incubated for 30 min at room temperature in the dark. The released glycine or taurine was measured at 416 nm using a SPARK 10M multimode microplate reader (TECAN, Männedorf, Switzerland). The tested conjugated bile salts were glycocholic acid (GCA, Sigma-Aldrich), glycochenodeoxycholic acid (GCDCA, Sigma-Aldrich), glycodeoxycholic acid (GDCA, Sigma-Aldrich), glycoursodeoxycholic acid (GUDCA, Tokyo Kasei Kogyo), taurocholic acid (TCA, Nacalai Tesque), taurochenodeoxycholic acid (TCDCA, Sigma-Aldrich), taurodeoxycholic acid (TDCA, Nacalai Tesque), and tauroursodeoxycholic acid (TUDCA, Sigma-Aldrich). Three independent experiments were performed (*n* = 24 each). Statistical analyses were performed using GraphPad Prism software (version 8.0; GraphPad Software, San Diego, CA, United States). The Student’s *t*-test was used, and a p-value less than 0.05 (*P* < 0.05) was considered statistically significant.

### Biochemical Characterization of LpBSH

The optimum enzymatic conditions of LpBSH were determined according to previous studies (Rani et al., 2017; Kusada et al., 2021) as follows. Taurodeoxycholic acid (TDCA) was selected as a representative substrate and mixed with 100 μg of purified LpBSH at various temperatures (10–90°C) and pH (pH 3.0–10.0) ranges. After incubation for 6 h, the released taurine was detected as described earlier. To determine the effects of pH on the enzyme activity of LpBSH, we used various Good’s buffer solutions based on the pH range (acetate buffer [CH_3_COONa.3H_2_O], pH 3.0–4.0; MES buffer [C_6_H_13_NO_4_S.H_2_O], pH 5.0–6.0; HEPES buffer [C_8_H_18_N_2_O_4_S], pH 7.0–8.0; and CAPS buffer [C_9_H_19_NO_3_S], pH 9.0–10.0). All experiments were performed with eight technical replicates.

### Antibiotics Susceptibility Test

Minimum inhibitory concentrations (MICs) were determined as the lowest concentration of antibiotics that prevented visible growth, as described previously (Kusada et al., 2017). In brief, an overnight full-growth culture of *L. paragasseri* JCM 5343^T^ was inoculated on MRS agar plates containing a selected β-lactam antibiotic. All plates were incubated at 37°C for 3 days under anaerobic conditions (AnaeroPouch, Mitsubishi Gas Chemical Co, Inc., Tokyo, Japan). The tested β-lactam antibiotics were penicillin G, penicillin V, ampicillin, amoxicillin, carbenicillin, cephalosporin C, cephalexin, and cefadroxil at final concentrations of 0.01, 0.05, 0.1, 0.5, 1.0, 5.0, 10.0, 20.0, 30.0, and 40.0 μg/ml. All experiments were performed in triplicates.

### Bile Salt Resistance Test

The assay was carried out to assess the resistance of *L. paragasseri* JCM 5343^T^ to bile salts, and survival rates were calculated according to a previous study (Gu et al., 2014). Briefly, a selected bile salt solution was added to an overnight full-growth culture of *L. paragasseri* JCM 5343^T^. Cells were anaerobically incubated at 37°C for 6 h, and the OD_600_ was measured every hour. Cells incubated in the GAM medium without bile salt were used as controls. The tested bile salts were GCA, GDCA, TCA, and TDCA at final concentrations of 0.1, 0.05, 0.1, and 0.1%, respectively. Note that the GDCA concentration was reduced to 0.05%, since a liquid GAM medium gradually becomes solid after adding 0.1% GDCA. All experiments were performed in triplicate.

In addition, the MICs were determined as the lowest concentration of the selected bile salts that completely prevented the visible growth of *L. paragasseri* JCM 5343^T^ on MRS agar. The full-grown culture of strain JCM 5343^T^ was inoculated onto MRS agar plates containing a selected bile salt. Plates were anaerobically incubated at 37°C for 5 days. The tested bile salts were GCA, GDCA, TCA, and TDCA at final concentrations of 0.1, 0.25, 0.5, and 0.75%. All experiments were performed in triplicate.

### Transcriptional Analyses of *lpBSH* Gene

Reverse transcription-polymerase chain reaction (RT-PCR) analyses of the *lpBSH* gene were performed as follows. Total RNA samples were isolated using the RNeasy Mini Kit (Qiagen, Germantown, MD, United States) from a full-grown culture of strain JCM 5343^T^ cultured in MRS broth with or without TDCA (final concentration of 0.05%) and cephalosporin C (final concentration of 10 μg/ml). To remove contaminating genomic DNA, the resulting RNA samples were treated with deoxyribonuclease (RT Grade) for Heat Stop (Nippon Gene Co., Ltd., Tokyo, Japan). The absence of contamination due to chromosomal genomic DNA was confirmed by PCR analysis using the 16S rRNA gene universal PCR primers 530F and 907R with each RNA sample as a template. According to the manufacturer’s instructions, reverse transcription reactions were performed using SuperScript IV Reverse Transcriptase (Thermo Fisher Scientific, Waltham, MA, United States). The synthesized complementary DNA (cDNA) samples were used as the PCR template with the following PCR primer sets: 5′-GTCGAGGTTTCAAAGCAATACGG-3′ and 5′-GCAGAATAGCAAGCAGTATAGACAG-3′, amplicon size: 388 bp. The PCR products were analyzed by 2% agarose gel electrophoresis and stained with GelRed (FUJIFILM Wako Pure Chemical Corporation).

### Conserved Amino Acid Sequences and Phylogenetic Analyses

We performed multiple amino acid sequence alignment analyses using the CLUSTAL W2 program and GENETYX-MAC software version 20.1.1 (GENETYX, Tokyo, Japan). The amino acid sequence of LpBSH was aligned with that of cysteine-nucleophile Ntn-hydrolase family proteins, including experimentally identified bifunctional enzymes (BsPVA, LgBSH, and CpBSH) and mono-functional BSH (EfBSH). In addition, a phylogenetic tree was constructed with MEGA X software using the neighbor-joining method (1,000 bootstrap replications) (Kumar et al., 2018). The phylogenetic tree was displayed and customized using the online tool Interactive Tree Of Life (iTOL v5)^6^ (Letunic and Bork, 2019). Amino acid sequences of experimentally identified cysteine-nucleophile Ntn-hydrolase proteins were used (all accession numbers are described in Supplementary Table 1).

### Structural Modeling of LpBSH

The three-dimensional conformation of LpBSH was predicted using the Swiss-Model workspace^7^ (Waterhouse et al., 2018). Superposition analyses were performed and visualized using UCSF Chimera software (Pettersen et al., 2004). The crystal structures of known BSH enzymes (CpBSH from *Clostridium perfringens* 13 (Rossocha et al., 2005) and EfBSH from *Enterococcus faecalis* T2 (Chand et al., 2018)) were obtained from the Protein Data Bank.^8^

## Results and Discussion

### Sequence Analyses of a Putative BSH Gene

**FIGURE 1:**
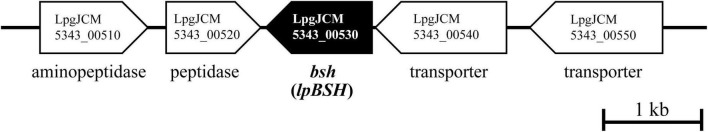
Physical map of the predicted bile salt hydrolase gene on the genome sequence of *L. paragasseri* JCM 5343^T^ (accession number: AP018549). The scale bar indicates a 1 kb length of nucleotide. A putative bile salt hydrolase gene (*lpBSH*) and other ORFs are represented by filled and open symbols, respectively. Locus tag ID and brief annotation are provided.

Based on the sequence analyses and homology search, we identified a putative bile salt hydrolase (*bsh*) gene (named *lpBSH*) in the *L. paragasseri* JCM 5343^T^ genome (Figure 1). Based on domain and sequence comparisons, the putative *bsh* was found to be related to cysteine-nucleophile Ntn-hydrolase family proteins. LpBSH showed a high amino acid sequence similarity to two characterized BSHs (93.67 and 93.04%) from *L. johnsonii* strains (Elkins and Savage, 1998; Oh et al., 2008), whereas it exhibited relatively low similarity to other identified BSHs (27.15–37.11%, 36.91–38.35%, and 32.91–34.69%) from *Lactiplantibacillus plantarum* strains (Christiaens et al., 1992; Ren et al., 2011; Gu et al., 2014), *Ligilactobacillus salivarius* strains (Fang et al., 2009; Wang et al., 2012; Bi et al., 2013), and *L. acidophilus* strains (McAuliffe et al., 2005), respectively. Furthermore, two putative bile salt transporter genes involved in the uptake of conjugated bile salts were identified downstream of the *lpBSH* gene (Figure 1). *bsh* and bile salt transporter genes probably constitute an operon (Elkins et al., 2001), which led this gene order to suggest that the *lpBSH* gene functions as a BSH. Notably, a BSH enzyme (BSH12) from *L. johnsonii* La1 has been reported to show no significant BSH activity, despite having high sequence similarity (∼99.08%) to the experimentally identified BSHs (Allain et al., 2018b). This observation suggested that only sequence-based functional prediction of the *bsh* gene was unreliable and further necessitates biochemical characterization to assess the *bsh* gene function (BSH activity) carefully and accurately.

The enzyme LpBSH exhibited low sequence similarity to the three experimentally identified PA enzymes, BsuPVA from *Bacillus subtilis* 168 (31.68%), PaPVA from *Pectobacterium atrosepticum* SCRI1043 (25.22%), and AtPVA from *Agrobacterium tumefaciens* ATCC 33970 (21.63%). Furthermore, we confirmed that LpBSH also showed relatively low sequence homology to the three experimentally identified bifunctional enzymes showing both BSH and PA activities: CpBSH from *Clostridium perfringens* 13 (38.36%), LgBSH from *Lactobacillus gasseri* FR4 (39.32%), and BsPVA from *Lysinibacillus sphaericus* ATCC 14577 (34.27%). Thus, although these sequence analyses suggested that LpBSH might have BSH activity, it remains unclear whether LpBSH also possessed PA activity. Therefore, we next performed cloning and heterologous expression of the gene and further characterized its biochemical features.

### Enzymatic Properties of a Recombinant LpBSH

**FIGURE 2:**
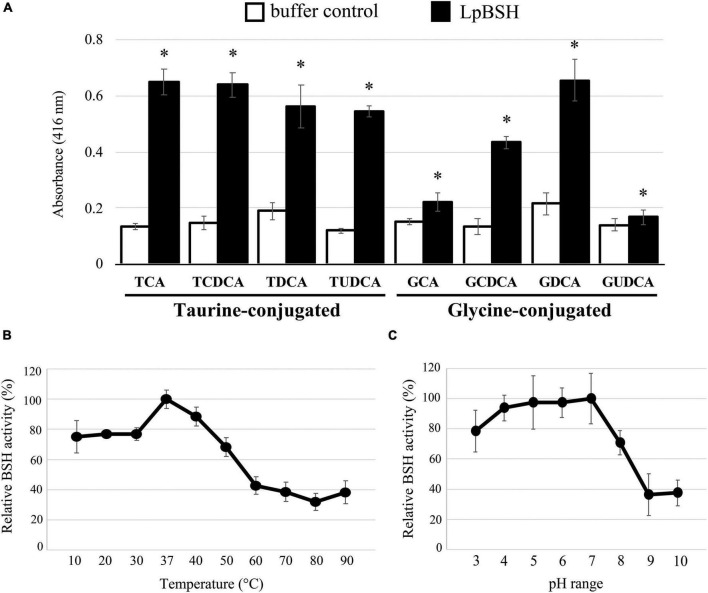
BSH activity and biochemical characterization of LpBSH. **(A)** BSH activity was measured against eight human bile salts: taurocholic acid (TCA), taurochenodeoxycholic acid (TCDCA), taurodeoxycholic acid (TDCA), tauroursodeoxycholic acid (TUDCA), glycocholic acid (GCA), glycochenodeoxycholic acid (GCDCA), glycodeoxycholic acid (GDCA), and glycoursodeoxycholic acid (GUDCA). Values are indicated as means for three independent experiments (*n* = 24, ^∗^*p* < 0.05). Error bars represent standard deviation (SD). Effects of temperature **(B)** and pH **(C)** on BSH activity against TDCA. Each value is expressed as mean values for eight technical replicates (each *n* = 8). Maximum activity was taken as 100%. Error bars indicate SD.

We characterized the BSH activity and substrate specificity of the recombinant LpBSH toward eight mammalian bile salts (TCA, TCDCA, TDCA, TUDCA, GCA, GCDCA, GDCA, and GUDCA; see Materials and Methods section) by detecting taurine or glycine released from the hydrolysis of conjugated bile salts. As shown in Figure 2A, the LpBSH protein exhibited significant hydrolysis activity toward all the conjugated bile salts tested. In particular, LpBSH showed high activity toward TCA, TCDCA, TDCA, TUDCA, GCDCA, and GDCA, whereas very weak activities were observed when GCA and GUDCA were used as substrates. Intriguingly, although previously identified BSHs prefer to hydrolyze glycine-conjugated bile salts rather than taurine-conjugated ones (Coleman and Hudson, 1995; Tanaka et al., 2000; Kim et al., 2004), our investigation revealed that LpBSH hydrolyzed taurine-conjugated bile salts and glycine-conjugated bile salts. Furthermore, LjBSHB from *L. johnsonii* PF01 (93.04% similarity to LpBSH) hydrolyzes only taurine-conjugated bile salts (TCA, TCDCA, TDCA, THDCA, and TUDCA) (Oh et al., 2008), indicating that LpBSH has different substrate specificity from LjBSHB, even though they share high sequence similarity. In contrast, LjBSHb from *L. johnsonii* 100–100 also has high similarity to LpBSH (93.67%) and LjBSHB (98.73%) and can deconjugate both glycine- and taurine-conjugated bile salts (GCA, GCDCA, TCA, TCDCA, and TDCA) (Lundeen and Savage, 1990). Lundeen and Savage (1990) further reported that LjBSHb showed the highest activity toward GCA among the five substrates tested (Lundeen and Savage, 1990), while LpBSH exhibited very low activity toward GCA compared to that of the other substrates (Figure 2A). These different substrate specificities indicated that the substrate preference of BSHs was enzyme-specific, regardless of their high amino acid sequence similarities. Furthermore, LpBSH could hydrolyze six major bile salts in the human gut (GCA, GCDCA, GDCA, TCA, TCDCA, and TDCA) and two minor bile salts (GUDCA and TUDCA), although it remained unclear whether most of the characterized BSHs hydrolyze these minor substrates. These findings indicated that LpBSH could act as a functional BSH enzyme with broad substrate specificity.

The effects of temperature and pH on the enzyme activity of LpBSH were determined as described in the Materials and Methods section. The maximum BSH activity of LpBSH was observed at 37°C (Figure 2B). LpBSH showed high BSH activity in the low- and mid-temperature range and retained more than 75% of its original activity at 10–40°C, while it showed a significant reduction in enzyme activity at temperatures higher than 50°C (Figure 2B). Since the enzymatic activities of known BSHs significantly declined at low-temperature ranges of 20–30°C (Oh et al., 2008; Chae et al., 2013; Rani et al., 2017), we hypothesized that LpBSH with relatively high activity at low temperatures could be unique. The highest BSH activity of LpBSH was observed at pH 7.0 (Figure 2C). This enzyme exhibited stable activity and retained approximately 80% of its original activity at pH 3.0–7.0, whereas a significant decrease in its enzyme activity was observed at a pH higher than 8.0 (Figure 2C). Dong and Lee (2018) reported that previously characterized BSHs from *Lactobacillus* species vary considerably at optimum temperatures (mainly 30–55°C) and optimum pH (ranging from 3.8 to 7.0) for their activity (Dong and Lee, 2018). Moreover, LpBSH exhibited different biochemical characteristics from the two BSHs (LjBSHB and LjBSHb) with high sequence similarity to LpBSH. It is known that the optimum pH of LjBSHB is 6.0, and its BSH activity rapidly declines below pH 5.0 (Oh et al., 2008), whereas LjBSHb acts optimally at a low pH range (pH 3.8–4.5), and its BSH activity decreases above pH 5.5 (Lundeen and Savage, 1990), suggesting that the biochemical characteristics of BSHs would also be different for each enzyme despite their high degree of sequence homologies. Collectively, evidence supports that the optimum enzymatic conditions of LpBSH (37°C and pH 7.0) are consistent with both human intestinal environments and favorable growth conditions of *L. paragasseri* strain JCM 5343^T^ (25–45°C, pH 4.0–8.0) (Tanizawa et al., 2018).

### Penicillin Acylase Activity of a Recombinant LpBSH

**FIGURE 3:**
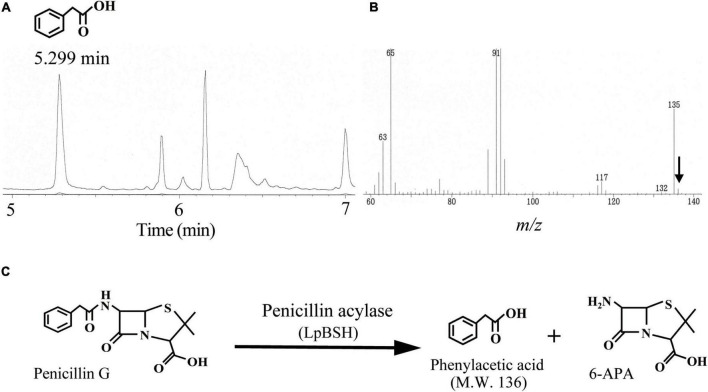
Gas chromatography-mass spectrometry analyses of penicillin G degradation products by LpBSH. Gas chromatography profile of digestion products of penicillin G by LpBSH **(A)** and its mass spectrometry retention times at 5.299 min **(B)**. The arrow indicates the parent peak (*m/z* 136) of the 5.299 min GC fraction. Schematic representation of penicillin G degradation by PA activity of LpBSH **(C)**.

To determine whether the recombinant LpBSH possesses PA activity, gas chromatography-mass spectrometry (GC-MS) analysis was performed to detect phenylacetic acid (molecular weight 136), which is generated by hydrolysis of penicillin G by PA activity (Kusada et al., 2017). We observed a significant peak with a GC retention time of 5.299 min when the recombinant LpBSH was mixed with penicillin G solution (Figure 3A). Mass spectrometry analyses of the 5.299 min GC fraction showed M-H ions at *m/z* 136 (Figure 3B), identical to the molecular weight of phenylacetic acid. In contrast, this peak was not detected when penicillin G solution was incubated with the buffer used (Supplementary Figure 2). These results indicate that recombinant LpBSH shows the PA activity that hydrolyzed the amide bond of penicillin G (Figure 3C). Therefore, we proposed the idea that since LpBSH is a bifunctional acylase capable of degrading β-lactam antibiotics together with conjugated bile salts, this enzyme might simultaneously provide ecological advantages (i.e., both antibiotic resistance and bile detoxification) for host *L. paragasseri* JCM 5343^T^ to enhance the survival rate in the mammalian intestine.

### Antibiotic Resistance and Bile Salt Degrading Ability of *L. paragasseri* JCM 5343^T^

We measured the minimal inhibitory concentrations (MICs) of eight different β-lactam antibiotics (penicillin G, penicillin V, ampicillin, amoxicillin, carbenicillin, cephalosporin C, cefalexin, and cefadroxil) against *L. paragasseri* JCM 5343^T^. The strain displayed a relatively high resistance profile to cephem-related β-lactam antibiotics (MICs were >10 μg/ml) compared to penicillin-related ones (0.05–0.5 μg/ml) (Table 1), despite the general susceptibility of *Lactobacillus* species to β-lactams (Zhou et al., 2005; Klare et al., 2007). Our previous study revealed that a PA enzyme (MacQ) confers β-lactam resistance to host bacterium by degrading β-lactams (Kusada et al., 2017; Yasutake et al., 2017). Although MacQ has no detectable amino acid sequence homology to LpBSH, these enzymes share the same enzymatic function of PA activity, suggesting that LpBSH may mediate β-lactam antibiotic resistance in *L. paragasseri* JCM 5343^T^.

**TABLE 1:** Minimum inhibitory concentrations of β-lactams against strain JCM 5343^T^.

Minimum inhibitory concentrations (μg/ml)							
**Penicillins**					**Chephems**		
	
PENG	PENV	AMP	AMO	CAR	CEC	CEP	CEF
0.05	0.1	0.1	0.1	0.5	>40	20	10

*Abbreviated as penicillin G (PENG), penicillin V (PENV), ampicillin (AMP), amoxicillin (AMO), carbenicillin (CAR), cephalosporin C (CEC), cephalexin (CEP), and cefadroxil (CEF). The tested concentrations of antibiotics were 0.01, 0.05, 0.1, 0.5, 1.0, 5.0, 10.0, 20.0, 30.0, and 40.0 μg/ml.*

**FIGURE 4:**
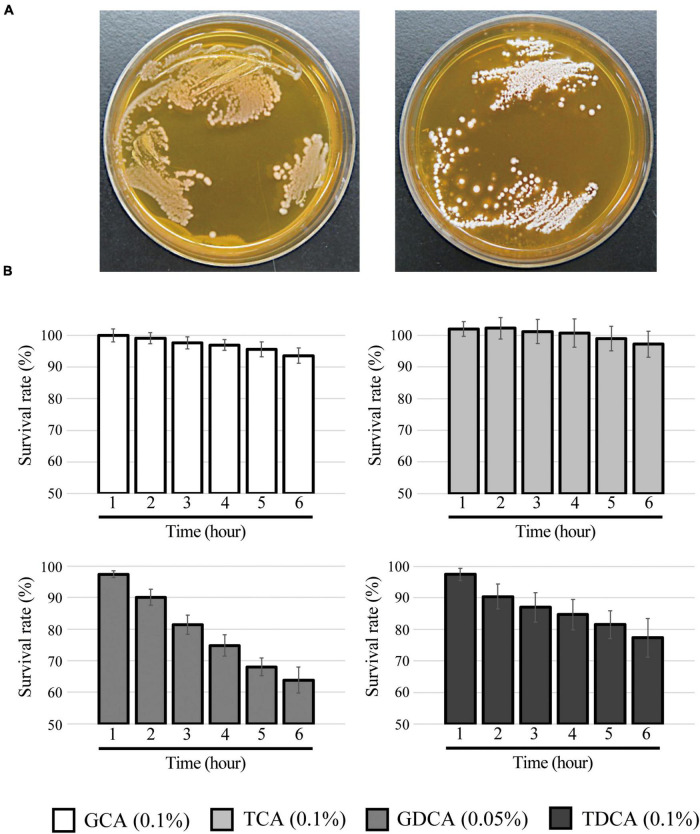
Bile salt hydrolase and bile salt resistance activities in *L. paragasseri* JCM 5343^T^. **(A)** Full-grown culture of *L. paragasseri* JCM 5343^T^ was streaked anaerobically on an MRS agar plate (left) or an MRS agar plate containing 0.25% TDCA (right). The plates were incubated at 37°C under anaerobic conditions for 3 days. **(B)** Overnight full-grown culture of *L. paragasseri* JCM 5343^T^ was inoculated into fresh GAM medium containing different bile salts (0.1% GCA, 0.05% GDCA, 0.1% TCA, and 0.1% TDCA) and incubated anaerobically at 37°C. The optimal density was measured every hour, and survival rates were assessed. The survival rate of control (without bile salt) was defined as 100%. Results indicated mean ± SD obtained in triplicate experiments.

Our investigations revealed that *L. paragasseri* JCM 5343^T^ also showed significant BSH capacity to hydrolyze conjugated bile salts by amidohydrolase activity. Indeed, we observed the heavy white precipitation of deconjugated bile salt surrounding *L. paragasseri* colonies [well-known indication of bacterial BSH activity (Sue et al., 2003)] on MRS agar plates supplemented with TDCA (Figure 4A). Intriguingly, some *Lactobacillus* species (e.g., *L. gasseri* CNCM I-4884 and *L. johnsonii* La1) with BSH activity have been reported to exhibit strong anti-parasitic activity against the well-known intestinal protozoan parasite *Giardia duodenalis* (Travers et al., 2016; Allain et al., 2018a). In addition, anti-*Giardia* activity of *Lactobacillus* species was positively correlated with the expression of BSH activities (Allain et al., 2018a). Therefore, we expected that a BSH-producing *L. paragasseri* JCM 5343^T^ would also show anti-parasitic activity, although further investigations are needed to uncover the additional probiotic (anti-parasitic) activity of this isolate. We further determined the survival rate of *L. paragasseri* JCM 5343^T^ against four different conjugated bile salts (TCA, TDCA, GCA, and GDCA). As shown in Figure 4B, *L. paragasseri* JCM 5343^T^ exhibited high survivability toward bile salts; in particular, the survival rates of strain JCM 5343^T^ were above 95% after exposure to the primary bile salts (GCA and TCA) for 6 h. In contrast, secondary bile salts (GDCA and TDCA) gradually decreased the survival rates of the strain (Figure 4B), since secondary bile salts are known to be more toxic than primary ones (Šušković et al., 2000). Moreover, we determined the MICs of four different bile salts for *L. paragasseri* JCM 5343^T^ (Table 2). As with the result of the survival rate analysis (Figure 4B), GDCA was the most toxic bile salt to *L. paragasseri* JCM 5343^T^, and the MIC of GDCA was 0.25%, whereas the strain displayed a much higher resistance activity against the other three bile salts (MICs were >0.75%) (Table 2).

**TABLE 2:** Minimum inhibitory concentrations of bile salts against strain JCM 5343^T^.

Minimum inhibitory concentrations (%)			
**Taurine-conjugated**		**Glycine-conjugated**	
	
TCA	TDCA	GCA	GDCA
>0.75	>0.75	>0.75	0.25

*Abbreviated as taurocholic acid (TCA), taurodeoxycholic acid (TDCA), glycocholic acid (GCA), and glycodeoxycholic acid (GDCA). The tested concentrations of bile salts were 0.1, 0.25, 0.5, and 0.75%.*

Given these physiological features, LpBSH seems to act as a protective mechanism not only against the toxicity of bile salts but also against the β-lactam antibiotics in *L. paragasseri* strain JCM 5343^T^. For future study, it would be necessary to test a deletion mutant of LpBSH in order to reach a definitive conclusion regarding how this bifunctional enzyme affects the strain’s ability as a probiotic. Bile salt resistance capacity is commonly recognized as an important biomarker for probiotic lactic acid bacteria. Furthermore, some beneficial effects of BSH-producing probiotic bacteria on human health have been elucidated previously [e.g., lipid digestion, changes in digestive functions, and serum cholesterol reduction (Begley et al., 2006)]. Future studies should examine the probiotic effect of BSH-producing *L. paragasseri* JCM 5343^T^ and its BSH enzymes (LpBSH) in an *in vivo* animal model study.

### Reverse Transcription PCR Analysis of *lpBSH* Gene in *L. paragasseri* JCM 5343^T^

**FIGURE 5:**
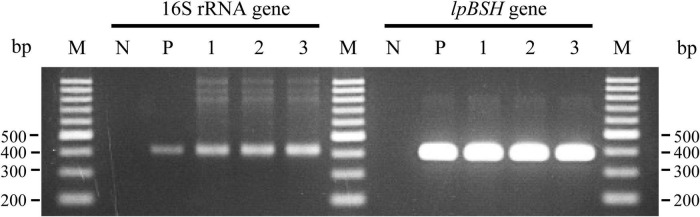
Reverse transcription-polymerase chain reaction analyses of *lpBSH* gene in *L. paragasseri* JCM 5343^T^. The synthesized cDNA samples from total RNA of non-supplemented (lane 1), TDCA-supplemented (lane 2), and cephalosporin C-supplemented (lane 3) strains of JCM 5343^T^ cells were used as the template for PCR analysis, respectively. Sterilized water (lane N) and genomic DNA of strain JCM 5343^T^ (lane P) were used as negative and positive control templates, respectively. The 16S rRNA gene was used as internal standard control. Lane M, molecular size markers (100 bp DNA ladder, Promega, Madison, WI, United States).

We investigated the transcription of the *lpBSH* gene in *L. paragasseri* JCM 5343^T^ using RT-PCR analysis. As shown in Figure 5, we found that the *lpBSH* gene was regularly transcribed in this strain (lane 1). We further observed that this gene was also transcribed in this strain after exposure to bile salts (TDCA, lane 2) or β-lactam antibiotics (cephalosporin C, lane 3). These results suggest that the presence of bile salts and β-lactam antibiotics would have little impact on *lpBSH* gene transcription in *L. paragasseri* JCM 5343^T^. Foley et al. (2021) recently reported that the transcription of *bsh* genes in *L. acidophilus* and *L. gasseri* differs according to the presence of bile salts (CA, GCA, and TCA), although drastic transcriptional changes have not been observed (Foley et al., 2021), suggesting that *bsh* gene transcription in *Lactobacillus* species might be regulated in a situation-dependent manner. Since both bile salts and β-lactams are widely known to be toxic to gut bacteria (Panda et al., 2014; Foley et al., 2019), it would be logical to consider that strain JCM 5343^T^ needs to constantly express the *lpBSH* gene and produce LpBSH protein in order to protect itself against both toxic bile salts and β-lactams and survive in the intestinal environment, though the degrees of effects of bile salts and antibiotics on the expression of *lpBSH* gene may vary.

### Multiple Alignment Analyses and Structural Modeling of LpBSH

Multiple amino acid sequence alignments of LpBSH with experimentally identified bifunctional enzymes (BsPVA, LgBSH, and CpBSH) and mono-functional BSH (EfBSH) revealed that the five residues (Cys-2, Arg-18, Asp-21, Asn-174, and Arg-227 in LpBSH) associated with the active site were completely conserved in all the proteins (Supplementary Figure 3). In particular, the N-terminal cysteine residue (Cys-2), known to play a critical role as a catalytic nucleophile in this family of proteins, was conserved (Rossocha et al., 2005). Meanwhile, seven amino acid residues (Met-20, Phe-26, Phe-61, Ala-68, Ile-133, Asn-136, and Leu-142) in CpBSH from *Clostridium perfringens* 13 (Rossocha et al., 2005) responsible for the potential substrate-binding were not fully conserved in the proteins (Supplementary Figure 3), suggesting that the lack of conservation in the amino acid sequence might cause significant differences in their substrate specificity. We further found three amino acid substitutions (Ile-53, Ala-114, and Thr-227) and two amino acid deficiencies (between Asp-10 and His-11) in EfBSH, BSH from *Enterococcus faecalis* T2 (Supplementary Figure 3). We hypothesized that since only EfBSH was not able to show PA activity (Chand et al., 2018) among the enzymes examined, amino acid residues (Met-56, Val-118, and Ala-232) conserved in all the other bifunctional BSHs including LpBSH and/or residues (Gly-12 and Asn-13) lacking in EfBSH might be associated with bifunctionality, although further experiments are warranted to verify this point.

Based on the 3D superposition analyses, we confirmed that the overall structure of LpBSH was composed of well-characterized αββα-sandwich folds (Supplementary Figure 4A), similar to the previously identified structure of CpBSH. LpBSH further shared a highly conserved catalytic active site structure (Cys, Arg, Asp, Asn, and Arg) with CpBSH (Supplementary Figure 4B). In addition, Chand et al. (2018) reported that four loop structures (loop1–loop4, these positions are shown in Supplementary Figure 3) of EfBSH were involved in the substrate specificity (Chand et al., 2018). All loop structures of EfBSH were superposed to LpBSH (Supplementary Figure 4C); however, we observed significant amino acid sequence variations among them. In particular, the amino acid residues involved in loop1, loop3, and loop4 of LpBSH were more hydrophobic than those of EfBSH. Since EfBSH is known as a mono-functional BSH enzyme unable to hydrolyze penicillin (Chand et al., 2018), these hydrophobic-rich loop structures might be attributable to the broad substrate specificity of LpBSH [e.g., it may enhance the substrate binding affinity and/or facilitate the coordination of substrates to the active site (Sunder et al., 2017)]. Our future work for additional crystallization and site-directed mutagenesis analyses of LpBSH will serve to verify its dual function mechanism in detail.

### Phylogenetic Analyses of Cysteine-Nucleophile Ntn-Hydrolase Proteins

**FIGURE 6:**
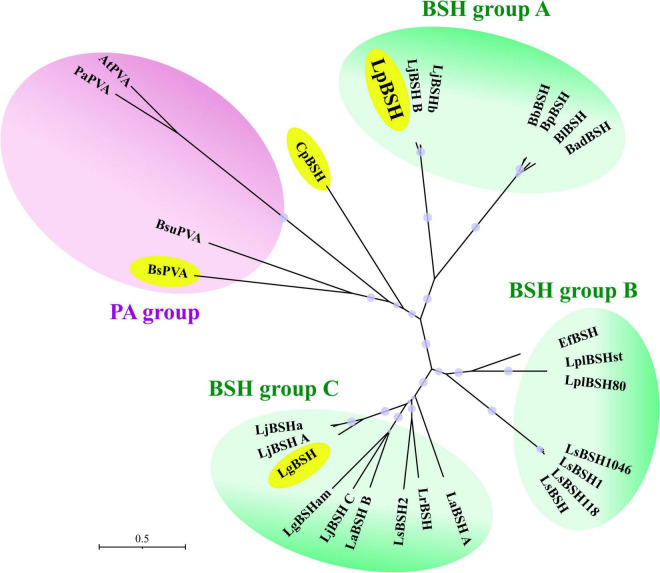
Phylogenetic analysis of LpBSH with cysteine-nucleophile Ntn-hydrolase proteins. Amino acid sequences of experimentally identified Ntn-hydrolase proteins were used in this analysis. Each enzyme name was simply defined based on the names of the host’s genus and species (e.g., LpBSH: *Lactobacillus paragasseri* derived BSH; for more information, please see Supplementary Table 1). The phylogenetic tree was constructed by the neighbor-joining method with MEGA X software (1,000 bootstrap replications). The evolutionary distances were computed using the Poisson correction method and are expressed as the units of the number of amino acid substitutions per site. The phylogenetic tree was divided into four groups [one PA group (purple background) and three BSH groups (green background)]. Four bifunctional acylases with PA and BSH activities (LpBSH, BuPVA, CpBSH, and LgBSH) were enclosed by yellow circles. Bootstrap values greater than 50% based on 1,000 replications are shown by circle symbols whose size correlates with the bootstrap values.

The Ntn-hydrolase family proteins were clearly divided into one PA group and three BSH groups (A, B, and C) (Figure 6). We found that EfBSH unable to exhibit PA activity was affiliated with BSH group B, and no bifunctional enzyme was found in this group to date, suggesting that BSHs in the BSH group B might not have bifunctionality. In contrast, we found that the identified bifunctional enzymes were widely distributed in the phylogeny (Figure 6). Moreover, LpBSH was clearly categorized into the BSH group A together with other experimentally characterized BSHs derived from intestinal bacteria. A well-known PA enzyme from *Lysinibacillus sphaericus* ATCC 14577 (BsPVA), which exhibits BSH activity, was categorized into the PA group. In addition, LgBSH from *Lactobacillus gasseri* FR4 has been reported to possess PA activity (Rani et al., 2017) and is affiliated with BSH group C. Although CpBSH from *Clostridium perfringens* 13 is also known to show PA activity (Rossocha et al., 2005), this bifunctional enzyme was not affiliated with any phylogenetic subgroup (Figure 6). This observation suggested that such bifunctionality would be broadly distributed among the Ntn-hydrolase family proteins and that bifunctional enzymes might be conserved in a wide variety of intestinal bacteria, including probiotic lactic acid bacteria. O’Flaherty et al. (2018) demonstrated that approximately 50% and 23% of *Lactobacillus* species (a total of 170 sequenced species) encoded putative PA proteins and putative BSH proteins, respectively (O’Flaherty et al., 2018). Our phylogenetic analysis revealed that some putative BSHs from *Lactobacillus* species were phylogenetically related to bifunctional LpBSH (e.g., accession numbers GL379581.1, KN050675.1, and KQ957048.1) and bifunctional LgBSH (MUJA01000002, AZCY01000001, and AFQJ01000004.1) (Supplementary Figure 5). This result further supported our hypothesis of the potential diversity and distribution of bifunctional enzymes. To summarize, our study is the first to discover the phylogenetic diversity and distribution of bifunctional BSH in intestinal microorganisms.

It has been reported that a PA enzyme (AtPVA) from *Agrobacterium tumefaciens*, a well-known aerobic Gram-negative plant pathogen, degrades *N*-acylhomoserine lactones (AHLs) by AHL acylase activity and attenuates AHL-mediated virulence in *Pseudomonas aeruginosa* (Sunder et al., 2017). Since AHL-acylase enzymes also belong to Ntn-hydrolase superfamily proteins similar to BSHs and PAs, we examined whether or not LpBSH possesses AHL-degradation activity by using AHL-detectable biosensor strains and GC-MS analysis as described previously (Kusada et al., 2017). However, LpBSH did not degrade any AHL compounds ranging from C_4_ to C_14_ chains with or without 3-oxo substitutions (data not shown). Importantly, AHLs are mostly produced and degraded by Gram-negative bacteria dwelling in aerobic environments, such as activated sludge, plant rhizosphere, microbial mat, and marine environments (Steidle et al., 2001; Huang et al., 2009; Shepherd and Lindow, 2009; Kusada et al., 2017; Liu et al., 2017), while neither AHL producers nor AHL degraders have been reported in Gram-positive bacteria isolated from anaerobic mammalian intestines (Swearingen et al., 2013). Since *L. paragasseri* JCM 5343^T^ is a Gram-positive bacterium isolated from the anaerobic human intestine and lacks the AHL synthase gene in the genome, our data on the substrate specificity of LpBSH are plausible.

On the other hand, BSH activity has been found primarily in the intestinal bacteria of several genera, including *Bacteroides*, *Bifidobacterium*, *Clostridium*, *Enterococcus*, and *Lactobacillus*. In contrast, no BSH activity has been observed in bacteria isolated from bile salt-free environments (Jones et al., 2008), except for a few recent cases (Yasiri et al., 2018; Nuhwa et al., 2019; Singhal et al., 2019). For instance, AtPVA and PaPVA (penicillin acylases) from *A. tumefaciens* and *Pectobacterium atrosepticum*, respectively, inhabiting plants and soils unlikely to contain bile salts did not show any BSH activity (Sunder et al., 2017; Daly et al., 2021). Hence, it has been proposed that BSH enzymes evolved to adapt to bile salts in intestine-specific environments (Begley et al., 2005; Jones et al., 2008). The recent overuse of antibiotics in clinical practice led us to hypothesize that BSH enzymes of intestinal bacteria might have acquired penicillin-degrading (penicillin acylase) activity during the process of adaptation to antibiotics in the digestive tract.

## Conclusion

In this study, we isolated and characterized a BSH enzyme (LpBSH) from *L. paragasseri* JCM 5343^T^. We further validated its bifunctional capability to degrade β-lactam antibiotics and conjugate bile salts by amidohydrolase activity. Although antibiotic resistance and bile detoxification have been recognized as distinct microbial functions, our findings reveal that the BSH enzyme could confer antibiotic resistance and bile salt resistance to *L. paragasseri* JCM 5343^T^. This further suggested that such a bifunctional BSH is likely able to provide a competitive advantage (high resistance to antibiotics and bile salts) to the host intestinal microorganisms, including probiotic lactic acid bacteria, since both conjugated bile salts and antibiotics are widely known to be toxic to intestinal bacteria. Furthermore, although the physiological and ecological effects of PA on host bacteria have been rarely investigated so far, this study proposed that the PA enzyme could be inextricably associated with the BSH enzyme responsible for several beneficial probiotic effects on human health (e.g., cholesterol-lowering effect). Hence, PA activity could be an overlooked new target for the probiotic function of intestinal bacteria. Collectively, our findings provide unprecedented insights into the physiological role of BSH and PA in intestinal microorganisms, especially probiotics.

## Data Availability Statement

The original contributions presented in study are included in the article/Supplementary Material, further inquiries can be directed to the corresponding authors.

## Author Contributions

HK, MA, MT, and HT: conceptualization and investigation. HK and HT: data curation, formal analysis, funding acquisition, supervision, and writing of the original draft. HT: project administration. All authors have read and agreed to the final version of the manuscript.

## Conflict of Interest

The authors declare that the research was conducted in the absence of any commercial or financial relationships that could be construed as a potential conflict of interest.

## Publisher’s Note

All claims expressed in this article are solely those of the authors and do not necessarily represent those of their affiliated organizations, or those of the publisher, the editors and the reviewers. Any product that may be evaluated in this article, or claim that may be made by its manufacturer, is not guaranteed or endorsed by the publisher.

## Footnotes

1
https://blast.ncbi.nlm.nih.gov/Blast.cgi

2
https://www.uniprot.org/blast/

3
http://www.ebi.ac.uk/interpro/search/sequence-search

4
http://pfam.xfam.org/

5
http://www.cbs.dtu.dk/services/SignalP/

6
https://itol.embl.de/

7
https://swissmodel.expasy.org/

8
http://www.rcsb.org/pdb/home/home.do
