# Supplementary material for: Bile Salt Hydrolase Degrades β-Lactam Antibiotics and Confers Antibiotic Resistance on Lactobacillus paragasseri
Source: Front Microbiol. 2022 Jun 6;13:858263. doi: 10.3389/fmicb.2022.858263 (PMC9207391; doi:10.3389/fmicb.2022.858263)
Supplement: Supplementary file 1 [file Table_1.DOCX]

Supplementary Material


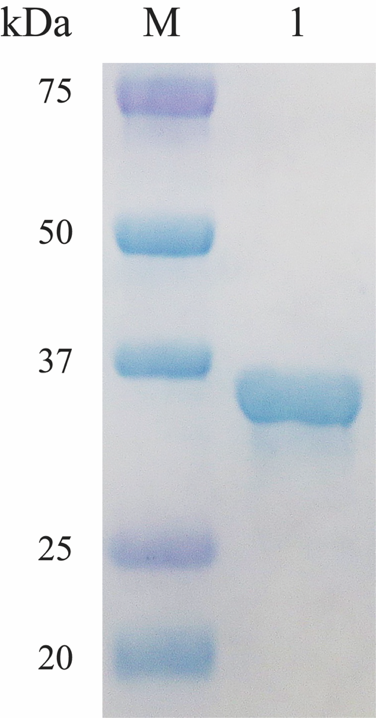


**Supplementary Figure 1.** Sodium dodecyl sulfate-polyacrylamide gel electrophoresis analysis of purified LpBSH. After nickel affinity chromatography, purified LpBSH protein was loaded onto a 12% SDS-PAGE gel. The single protein band of purified His_6_-LpBSH protein was observed to be around 37 kDa, which is nearly consistent with the calculated molecular weight using that of His_6_ (0.8 kDa) and LpBSH (35 kDa). Lane M, molecular size-marker; lane 1, LpBSH.


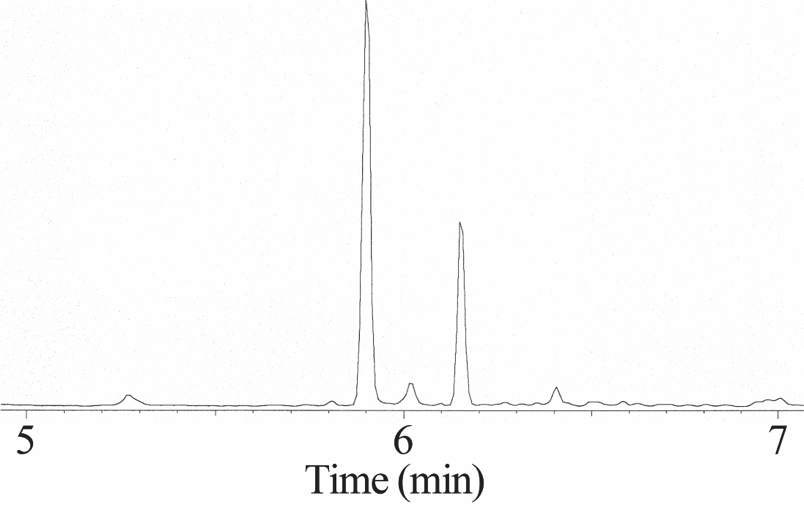


**Supplementary Figure 2.** Gas chromatography profile of intact penicillin G solution with control buffer.


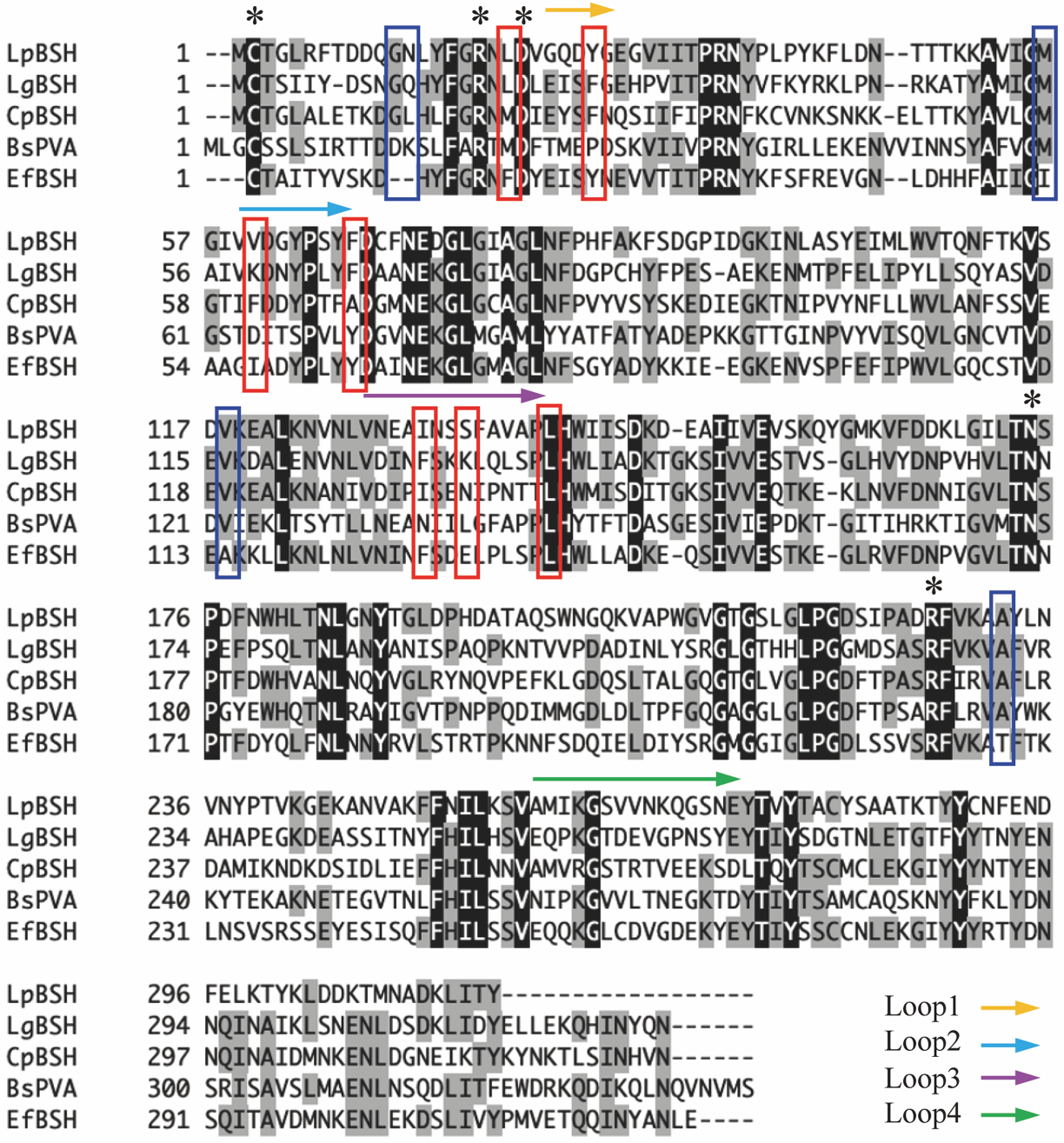


**Supplementary Figure 3.** Multiple alignment of amino acid sequences of LpBSH and other cysteine-nucleophile Ntn-hydrolase proteins. The black and gray shading indicates identical and similar amino acid residues, respectively. The conserved residues (Cys, Arg, Asp, Asn, and Arg) relevant to the predicted active site are indicated by black asterisk. Amino acid residues involved in substrate binding are boxed in red. Amino acid residues which are not conserved in mono-functional EfBSH are boxed in blue. Abbreviated as: LpBSH (BBD47700) from *Lactobacillus paragasseri* JCM 5343^T^; LgBSH (WP_020806888) from *Lactobacillus gasseri* FR4; CpBSH (P54965) from *Clostridium perfringens* 13; BsPVA (P12256) from *Lysinibacillus sphaericus* ATCC 14577; EfBSH (4WL3) from *Enterococcus faecalis* T2.


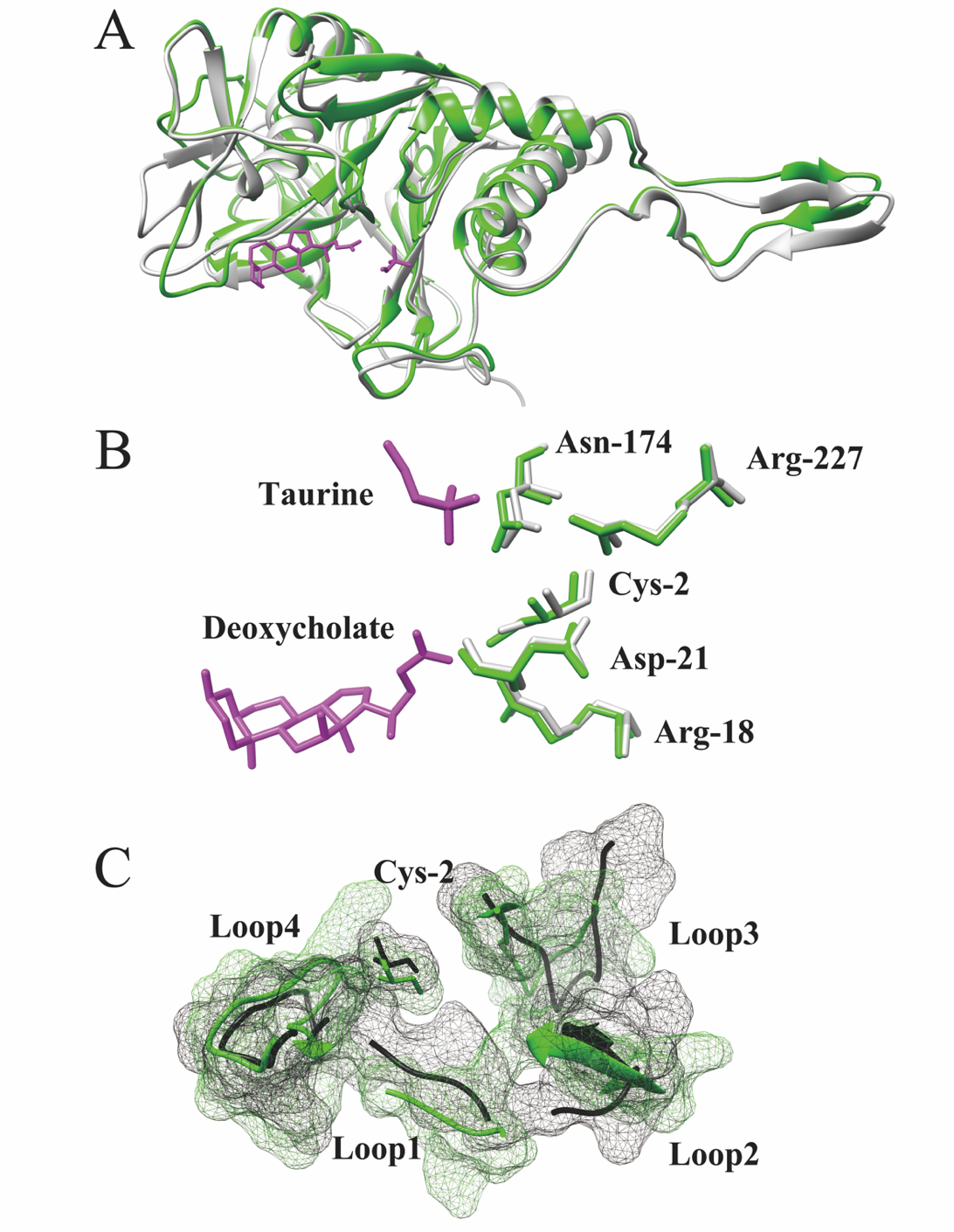


**Supplementary Figure 4.** Structural analyses of LpBSH. Overall (A) and active site (B) structural superposition of LpBSH with CpBSH, BSH from *Clostridium perfringens* 13 (Rossocha, et al., 2005) (grey; PDB entry 2BJF). Taurine and deoxycholate molecules from CpBSH were shown in magenta stick. (C) Comparison of the loop structures of LpBSH with EfBSH, BSH from *Enterococcus faecalis* T2 (Chand, et al., 2018) (black; PDB entry 4WL3).


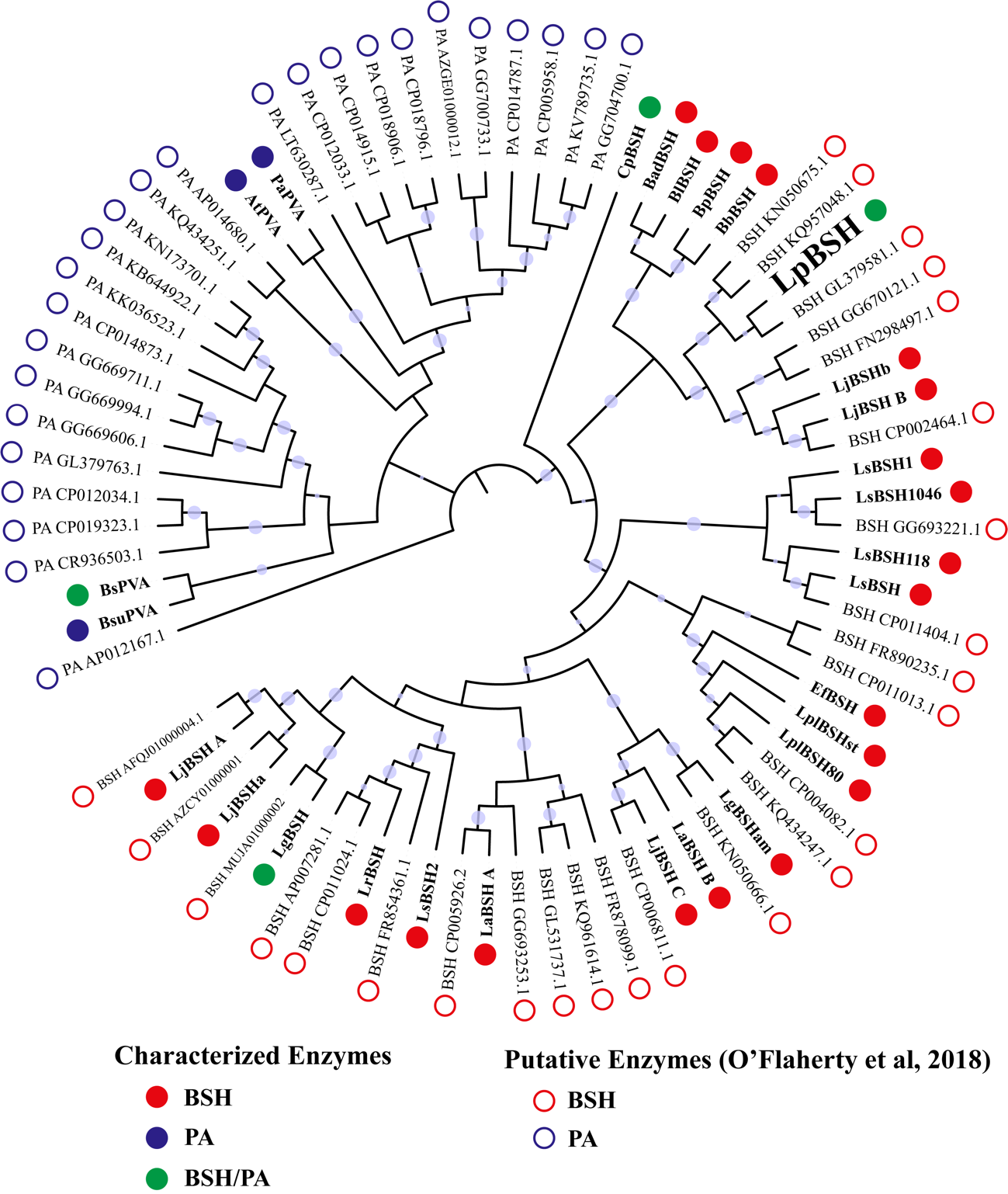


**Supplementary Figure 5.** Phylogenetic analysis of putative PAs and BSHs. Amino acid sequences of putative PAs and BSHs were obtained from a previous study (O'Flaherty et al., 2018) and indicated by open circles. Amino acid sequences of experimentally identified PAs, BSHs, and bifunctional enzymes were indicated by filled circles. The phylogenetic tree was constructed with MEGA X software using the neighbor joining method (1,000 bootstrap replications). Bootstrap values greater than 50% based on 1,000 replications are shown by circle symbols whose size correlates with the bootstrap values.

**Supplementary Table 1.** Amino acid sequences of experimentally identified Ntn-hydrolase proteins used in this analysis.

| **Abbreviation** | **Accession number** | **Strain** |
| --- | --- | --- |
| LpBSH | BBD47700 | *Lactobacillus paragasseri* JCM 5343^T^ |
| AtPVA | 5J9R | *Agrobacterium tumefaciens* ATCC 33970 |
| PaPVA | 4WL2 | *Pectobacterium atrosepticum* SCRI1043 |
| BsPVA | P12256 | *Lysinibacillus sphaericus* ATCC 14577 |
| BsuPVA | 2OQC | *Bacillus subtilis* 168 |
| CpBSH | P54965 | *Clostridium perfringens* 13 |
| LjBSHA | EGP12224 | *Lactobacillus johnsonii* PF01 |
| LjBSHB | ABQ01980 |  |
| LjBSHC | EGP12391 |  |
| LjBSHa | AAG22541 | *Lactobacillus johnsonii* 100-100 |
| LjBSHb | AAC34381 |  |
| LaBSHA | AAV42751 | *Lactobacillus acidophilus* NCFM |
| LaBSHB | AAV42923 |  |
| LgBSH | WP_020806888 | *Lactobacillus gasseri* FR4 |
| LgBSHam | ACL98172 | *Lactobacillus gasseri* AM1 |
| LrBSH | WP_035157795 | *Limosilactobacillus reuteri* CRL1098 |
| LsBSH1 | ACL98197 | *Ligilactobacillus salivarius* LGM 14476 |
| LsBSH2 | ACL98205 |  |
| LsBSH118 | ACL98201 | *Ligilactobacillus salivarius* UCC118 |
| LsBSH1046 | ACL98194 | *Ligilactobacillus salivarius* JCM 1046 |
| LsBSH | AFP87505 | *Ligilactobacillus salivarius* B-30514 |
| LplBSHst | ADO00098 | *Lactiplantibacillus plantarum* subsp. *plantarum* ST-III |
| LplBSH80 | AAB24746 | *Lactiplantibacilluss plantarum* 80 |
| EfBSH | 4WL3 | *Enterococcus faecalis* T2 |
| BbBSH | AAR39435 | *Bifidobacterium bifidum* ATCC 11863 |
| BlBSH | AAF67801 | *Bifidobacterium longum* SBT2928 |
| BadBSH | AAX86039 | *Bifidobacterium adolescentis* ATCC 15705 |
| BpBSH | KFI75916 | *Bifidobacterium pseudocatenulatum* DSM 20438 |
